# The Effects of Sirolimus and Magnesium on Primary Human Coronary Endothelial Cells: An In Vitro Study

**DOI:** 10.3390/ijms24032930

**Published:** 2023-02-02

**Authors:** Giorgia Fedele, Sara Castiglioni, Jeanette A. M. Maier, Laura Locatelli

**Affiliations:** Department of Biomedical and Clinical Sciences, Università di Milano, 20157 Milano, Italy

**Keywords:** sirolimus, hCAEC, magnesium

## Abstract

Drug eluting magnesium (Mg) bioresorbable scaffolds represent a novel paradigm in percutaneous coronary intervention because Mg-based alloys are biocompatible, have adequate mechanical properties and can be resorbed without adverse events. Importantly, Mg is fundamental in many biological processes, mitigates the inflammatory response and is beneficial for the endothelium. Sirolimus is widely used as an antiproliferative agent in drug eluting stents to inhibit the proliferation of smooth muscle cells, thus reducing the occurrence of stent restenosis. Little is known about the potential interplay between sirolimus and Mg in cultured human coronary artery endothelial cells (hCAEC). Therefore, the cells were treated with sirolimus in the presence of different concentrations of extracellular Mg. Cell viability, migration, barrier function, adhesivity and nitric oxide synthesis were assessed. Sirolimus impairs the viability of subconfluent, but not of confluent cells independently from the concentration of Mg in the culture medium. In confluent cells, sirolimus inhibits migration, while it cooperates with Mg in exerting an anti-inflammatory action that might have a role in preventing restenosis and thrombosis.

## 1. Introduction

Cardiovascular diseases, in particular coronary heart disease (CAD) and stroke, rank as the first global cause of death [1]. CAD, which is the most common type of heart disease, is mainly due to the presence of atherosclerotic plaques that narrow the lumen of the coronary segments, thus reducing blood flow to the heart. Percutaneous coronary intervention, previously known as angioplasty with stenting, is a minimally invasive procedure that relieves the stenosis and provides mechanical support to the vessel wall [2]. Starting from the generation and first implantation of bare metal stents, many efforts have been expended to improve the outcome of patients and to restore vessel function with the fewest adverse effects as possible, reducing the risk of in-stent restenosis (ISR), thrombosis and inflammatory reactions [3]. Drug Eluting Stents represent a second generation of implants that release anti-proliferative and immunosuppressive drugs to prevent the neointimal hyperplasia, a crucial event for ISR [4]. The delivered drugs are usually the mammalian target of rapamycin (mTOR) inhibitors such as sirolimus (SIR, also known as rapamycin) and its derivatives, which impair smooth muscle cells’ (SMC) proliferation and migration [5]. It should be noted that, while inhibiting cell migration is an asset in preventing neointimal growth, it also leads to impaired stent endothelialization. Moreover, the presence of a foreign body is the trigger of chronic inflammation, delayed endothelialization, impaired arterial healing and neoatherosclerosis [6]. A new generation of stents made with Bioresorbable Scaffolds (BRS) has emerged. BRS eluting an anti-proliferative drug and degrading over time have attracted a lot of attention [7], and several clinical trials have confirmed their safety [8]. Drug eluting magnesium (Mg) resorbable scaffolds are attractive since novel Mg-based alloys such as WE43/Mg-Y-RE-Zr [9,10] have adequate mechanical properties and are resorbed without adverse events [11]. In addition, Mg, which is involved in many physiological processes and is crucial for several vital functions [12,13,14], is beneficial for blood vessels. While low Mg is associated with endothelial dysfunction and atherosclerosis [15], high Mg levels prevent oxidative stress and inflammation in endothelial cells [14,16]. Moreover, Mg has a role in promoting endothelial migration and proliferation [17] and, therefore, might favor the process of re-endothelialization [18].

Since rabbits are commonly used as a preclinical model of drug eluting scaffolds, we analyzed the interplay between SIR and Mg in rabbit-cultured coronary endothelial and smooth muscle cells, and showed that Mg contributes to the maintenance of endothelial function without interfering with the effects of SIR on SMC [12]. Importantly, Mg counterbalances the inhibitory effects of SIR on endothelial migration. Since Mg resorbable scaffolds have been implanted also in humans with challenging results [19,20,21], we investigated the possible reciprocal interactions between Mg and SIR in primary human coronary endothelial cells (hCAEC), an issue that has not been previously addressed. 

## 2. Results

### 2.1. The Effects of Mg and SIR on hCAEC Viability

**Figure 1:**
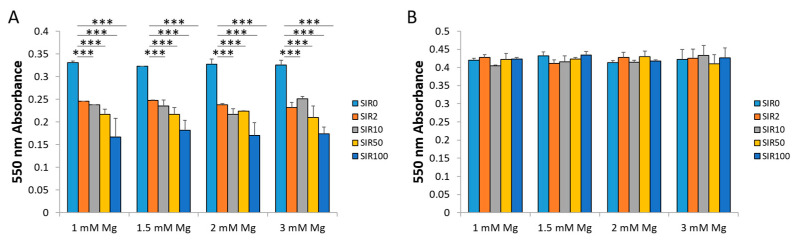
The effects of SIR on the viability of hCAEC. Subconfluent (**A**) and confluent (**B**) hCAEC were treated with different concentrations of SIR (0, 2, 10, 50, 100 ng/mL) in the presence of 1, 1.5, 2 or 3 mM Mg. After 48 h of treatment, an MTT assay was performed, and absorbance was measured at 550 nm. Data are expressed as the mean ± standard deviation (SD) of three separate experiments in triplicate (*** *p* ≤ 0.001).

We tested whether hCAEC viability was modulated by the presence of different concentrations of SIR (0, 2, 10, 50 and 100 ng/mL) in combination with Mg (1, 1.5, 2, 3 mM) for 48 h. The concentrations used were selected based on studies performed in preclinical models [22]. We performed our experiments utilizing two cell densities. Subconfluent cells were used because, after stent implantation, endothelial cells are sparse and proliferate to repopulate the injured vascular wall. Confluent hCAEC were analyzed because the cells neighboring the lesion form a continuous layer that might be exposed to the products of stent degradation. Subconfluent cells are deeply affected by the presence of SIR even at very low concentrations, and Mg does not prevent the decrease in cell viability (Figure 1A). On the contrary, when the cells are confluent, they are resistant to the drug, also at very high concentrations (Figure 1B). No differences were found between the different concentrations of both SIR and Mg. On these bases, we decided to continue our experiments selecting some concentrations that represent both high and low condition of treatment: SIR 2 ng/mL (SIR2), a low concentration that exerts biological effects, and 50 ng/mL (SIR50), the maximum concentration of SIR measured in the vessel wall immediately after stent implantation [23,24]. As for Mg concentrations, we utilized medium containing 1 mM Mg, which is the physiological concentration, and 3 mM Mg, which reflects the amounts of Mg accumulating in the vessel after implantation in preclinical models [22]. 

### 2.2. The Effects of Mg and SIR on hCAEC Cytoskeletal Organization and Mg^2+^ Intracellular Content 

**Figure 2:**
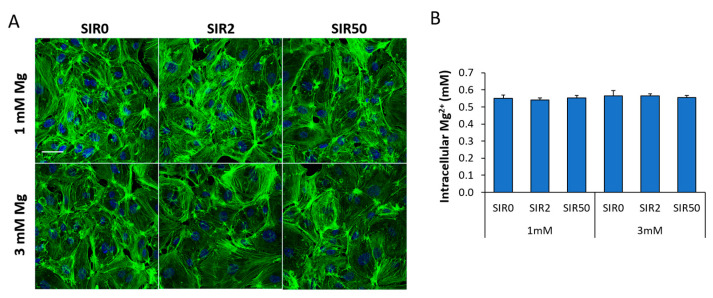
hCAEC cytoskeletal organization and Mg^2+^ levels in response to SIR and Mg. hCAEC were cultured with 1 or 3 mM Mg for 48 h in the absence (SIR0) or in the presence of SIR (2 and 50 ng/mL). (**A**) The cells were stained with fluorescein phalloidin, and images were acquired using a 40× objective in oil by an SP8 Leica confocal microscope. Scale bar: 40 µm. (**B**) Intracellular Mg^2+^ was measured. Data are expressed as the mean ± SD of three separate experiments in duplicate.

We then evaluated the effects of the different treatments on cell shape and cytoskeletal organization, both markers of cellular health. After staining with fluorescent phalloidin, no alteration in cell morphology and cytoarchitecture was observed after 48 h of the different treatments (Figure 2A). In the same experimental conditions, we assessed the intracellular Mg^2+^ levels, without finding differences between samples (Figure 2B). 

### 2.3. The Effects of Mg and SIR on hCAEC Migration

**Figure 3:**
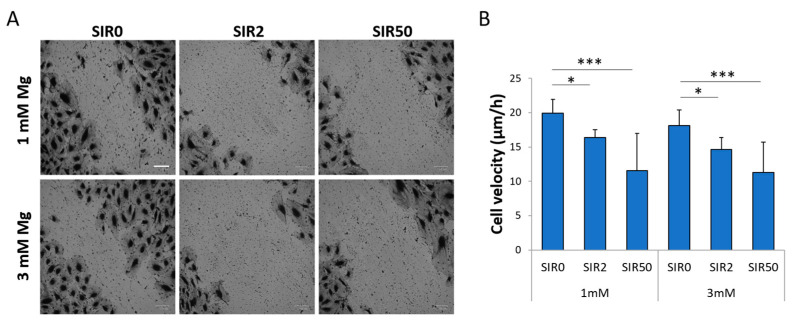
hCAEC migration in response to SIR. hCAEC were cultured with 1 or 3 mM Mg for 48 h in the absence (SIR0) or in the presence of SIR (2 and 50 ng/mL). (**A**) The wound assay was performed, and the cells were stained with Crystal Violet. Images were acquired by optical microscope (4× magnification). Scale bar: 50 µm. (**B**) The images were analyzed using ImageJ, and the results are expressed as the cell velocity (μm/h) ± SD. * *p* ≤ 0.05; *** *p* ≤ 0.001.

Since both SIR and Mg are known to have an impact on cell migration [12], we performed a scratch assay. Migration was inhibited by SIR in the presence of both 1 and 3 mM Mg (Figure 3). Moreover, 3 mM Mg alone did not affect hCAEC migration.

### 2.4. The Effects of Mg and SIR on the Interaction between hCAEC and Monocyte-Like Cells

**Figure 4:**
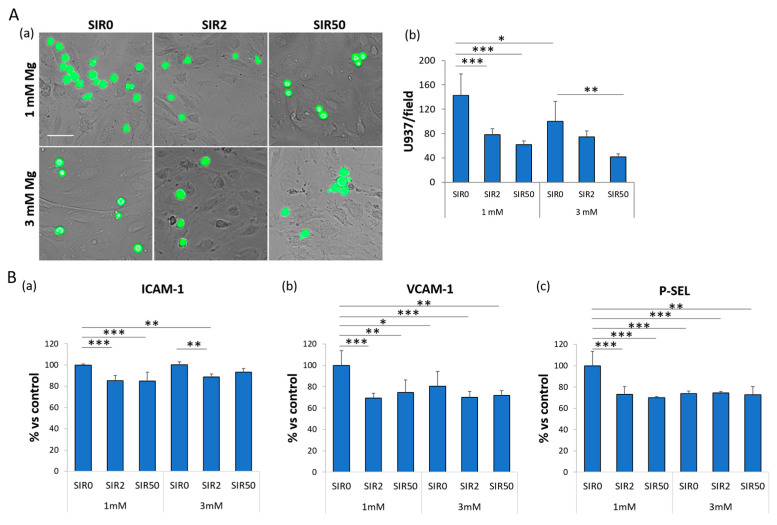
The effects of SIR on the adhesion of U937 to hCAEC. After 4 h of treatment with LPS, hCAEC were cultured with 1 or 3 mM Mg in the absence (SIR0) or in the presence of SIR (2 and 50 ng/mL). (**A**) (**a**) Fluorescent-U937 cells were incubated with hCAEC for 30 min. Images were acquired using Floid Microscope and the attached U937 for each field were counted. Scale bar: 40 µm. (**b**) The experiment was performed three times and the data are expressed as the number of cells attached/field ± SD. (**B**) ELISA assays for ICAM-1 (**a**), VCAM-1 (**b**) and P-SEL (**c**) were performed in triplicate for three times. Data are expressed as the percentage of the control (1 mM Mg SIR0) ± SD. * *p* ≤ 0.05; ** *p* ≤ 0.01; *** *p* ≤ 0.001.

Since inflammation is crucial for stent complications (i.e., ISR), we tested whether SIR might affect the adhesion of monocyte-like U937 cells to the endothelial layer. It is well known that the adhesion of leukocytes to the endothelium is an early event in inflammation and atherosclerosis [25,26,27,28]. Since under normal conditions endothelial cells do not bind leukocytes, the cells were treated with lipopolysaccharides (LPS) for 4 h to induce an inflammatory phenotype. LPS significantly increase hCAEC/U937 interaction, and this is prevented by culture in 3 mM Mg (Figure 4A and Figure S1). Moreover, SIR2 and SIR50 decrease the adhesivity of LPS-treated endothelial cells cultured in 1 mM Mg and do not influence the inhibitory action of 3 mM Mg.

To explain these results, we evaluated the expression of different adhesion molecules known to be involved in the recruitment of immune cells during the inflammatory response, i.e., Intercellular Adhesion Molecule 1 (ICAM-1), vascular cell adhesion molecule 1 (VCAM-1) and P-Selectin (P-SEL) (Figure 4B). By ELISA, we found that (i) 3 mM Mg alone suffices to significantly decrease the levels of VCAM-1 and P-SEL (Figure 4B(b,c)), and (ii) SIR downregulates the levels of ICAM-1, VCAM-1 and P-SEL compared to the control (1 mM Mg SIR0) (Figure 4B(a–c)).

### 2.5. The Effects of Mg and SIR on Endothelial Permeability

**Figure 5:**
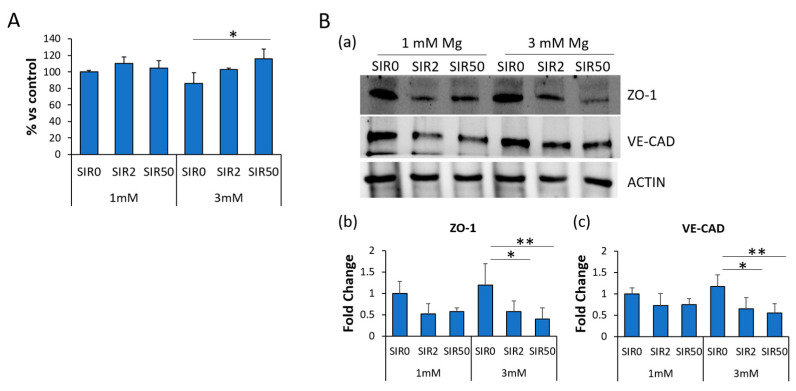
The effects of SIR on endothelial permeability. hCAEC were cultured with 1 or 3 mM Mg for 48 h in the absence (SIR0) or in the presence of SIR (2 and 50 ng/mL). (**A**) X-PerT Assay was performed, and fluorescence was read using Varioskan Lux (Thermo Fisher). The experiment was performed three times in triplicate. Data are expressed as the percentage to the control (1 mM Mg SIR0) ± SD. (**B**) Western blots on protein lysates using antibodies against ZO-1 and VE-CAD were performed. Anti-β-actin antibodies were used as control of equal loading. (**a**) A representative blot of three independent experiments is shown. (**b**,**c**) Densitometry of the bands was performed with the software ImageLab and expressed as the fold change compared to the control (1 mM Mg SIR0) ± SD. * *p* ≤ 0.05; ** *p* ≤ 0.01.

Dysregulated endothelial permeability plays a role in CAD [29,30,31]. Therefore, we measured the permeability of a monolayer of hCAEC treated with SIR for 48 h in the presence of 1 or 3 mM Mg using the X-PerT assay. A slight, although not significant, reduction in permeability was observed after exposure to 3 mM Mg, whereas SIR50 increased it. No significant differences were detected in 1 mM Mg with or without SIR (Figure 5A). Next, we investigated the amounts of two adhesion molecules known to be implicated in governing endothelial permeability. We focused on Zonula Occludens 1 (ZO-1), a member of the tight junction family, and Vascular Endothelial Cadherin (VE-CAD), a member of the adherens junction family. By western blot, we found that SIR tends to decrease the levels of both ZO-1 and VE-CAD, mostly in the presence of 3 mM Mg (Figure 5B). 

### 2.6. The Effects of Mg and SIR on the Localization of ZO-1 and VE-CAD

**Figure 6:**
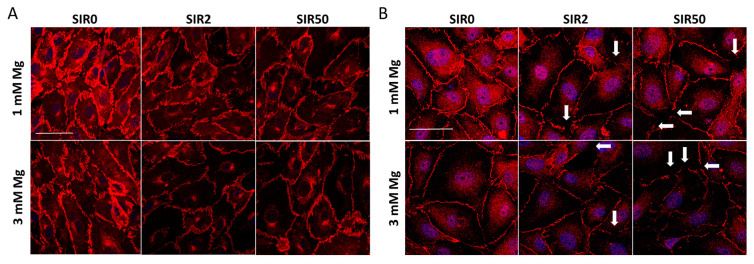
The effects of SIR on the localization of VE-CAD and ZO-1. hCAEC were cultured with 1 or 3 mM Mg for 48 h in the absence (SIR0) or in the presence of SIR (2 and 50 ng/mL). The cells were incubated with antibodies anti-VE-CAD (**A**) or anti-ZO-1 (**B**) and then stained with an Alexa Fluor 546 secondary antibody. DAPI was used to stain the nuclei. Images were acquired using a 40× objective in oil by an SP8 Leica confocal microscope. Scale bar: 40 µm.

To control permeability, the aforementioned adhesion molecules need to be properly localized on the membrane, creating a linear and continuous boundary between the cells. For this reason, we looked at VE-CAD (Figure 6A and Figure S2) and ZO-1 (Figure 6B and Figure S3) localization by immunofluorescence. SIR seems to affect only the amounts of VE-CAD (Figure 5B and Figure 6A), while it affects both the levels and the localization of ZO-1 (Figure 5B and Figure 6B). Indeed, upon the treatment with SIR, different interendothelial gaps were observed (white arrows in Figure 6B). 

### 2.7. The Effects of Mg and SIR on Nitric Oxide Production

**Figure 7:**
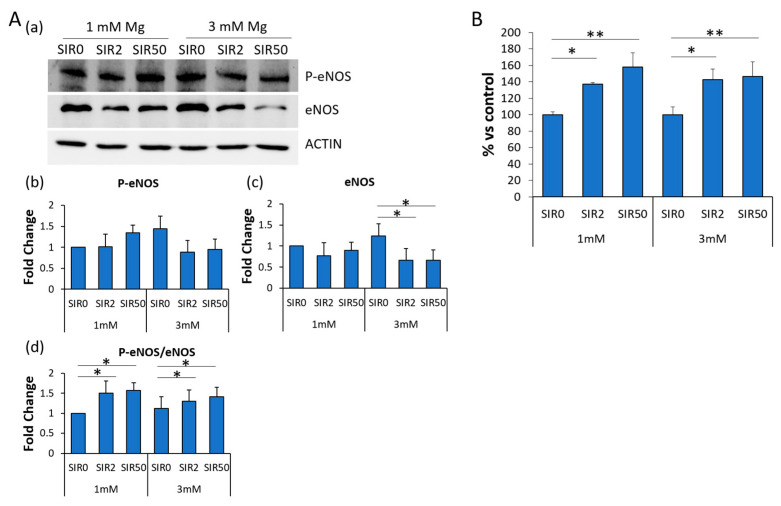
The effects of SIR on nitric oxide production hCAEC were cultured with 1 or 3 mM Mg for 48 h in the absence (SIR0) or in the presence of SIR (2 and 50 ng/mL). (**A**) Western blots on protein lysates using antibodies against eNOS and P-eNOS were performed. Anti-β-actin antibodies were used as control of equal loading. (**a**) A representative blot of three independent experiments is shown. (**b**,**c**) Densitometry of the bands was performed with the software ImageLab and expressed as the fold change compared to the control (1 mM Mg SIR0) ± SD. (**d**) Ratio P-eNOS vs. total eNOS was reported. (**B**) Released NO was measured using the Griess Assay. The experiment was performed three times in triplicate. Data are expressed as the percentage to the control (1 mM Mg SIR0) ± SD. * *p* ≤ 0.05; ** *p* ≤ 0.01.

Nitric oxide (NO) is crucial in maintaining endothelial integrity. It plays a role in regulating permeability and inflammatory responses. Because the inducible form of the enzyme (iNOS) is not expressed in our experimental conditions, we investigated whether the treatment with SIR might affect the expression of endothelial nitric oxide synthase (eNOS), constitutively expressed by endothelial cells but functionally regulated by a myriad of different factors. By western blot, we found that the treatment with SIR decreased the amounts of eNOS (Figure 7A(a,c)), and this inhibitory effect is more evident in hCAEC cultured in 3 mM Mg and SIR. However, the phosphorylated form (Ser1177) of eNOS (P-eNOS), which has higher enzymatic activity, remains unchanged (Figure 7A(a,b)), and the ratio P-eNOS vs. total eNOS is enhanced in SIR-treated cells (Figure 7A(d)). We then measured NO in the media and found that SIR enhances the release of NO (Figure 7B).

## 3. Discussion

Since Mg is an essential mineral for humans and maintains vascular integrity, Mg-based stents are highly biocompatible and might also drive healing in the injured vessel wall [18]. Novel alloys and coatings have increased Mg mechanical strength, plasticity and long term durability [4,9,10]. SIR eluting Mg-based scaffolds have been generated and shown to induce healing and reduce neo-atherosclerosis in rabbits [32]. These devices also show low thrombogenicity in rabbits and pigs [32]. Our results on cultured rabbit coronary endothelial cells and SMC explained, in part, the positive data obtained in preclinical animal models because Mg (3 mM) in the presence of SIR maintains normal endothelial proliferation and function, without affecting SMC growth inhibition [12].

These scaffolds were implanted in CAD patients, and clinical trials have yielded promising results [4]. However, in vitro evidence of the potential interaction of SIR and local increase of Mg as well as data on the effects of concentrations of Mg higher than the physiological one are lacking, especially on hCAEC. Here, we show that even a modest increase of extracellular Mg (3 mM) reduces the amounts of VCAM-1 and P-SEL, typically overexpressed in inflammation, resulting in a reduced adhesivity towards U937 cells. Moreover, SIR downregulates ICAM-1, VCAM-1 and P-SEL in hCAEC cultured in 1 mM Mg. These results are in agreement with a previous report showing that SIR prevents adhesion of leukocytes to activated TNFα-activated hCAEC in vitro through the downregulation of ICAM-1 and VCAM-1 [33]. The reduced accumulation of leukocytes represents an important step in preventing clinical restenosis. This event might be explained by the inhibition of NFkB pathway by SIR. Accordingly, in cultured human umbilical vein endothelial cells, rapamycin inhibits NFκB activation by reducing mTOR phosphorylation [34]. 

NO is fundamental for vascular homeostasis, and eNOS activity is an indicator of healthy endothelium. In hCAEC, SIR downregulates eNOS while its activating phosphorylated form remains unaffected, resulting in an increased ratio of P-eNOS vs. total eNOS. In parallel, SIR-treated cells release more NO than controls. These results are relevant especially if they are confirmed in vivo because maintaining or increasing NO is essential in preventing leukocyte adhesion, platelet aggregation and vascular tone [35]. For this purpose, it is known that genetic predisposition to potentiate NO signaling is associated with reduced risk of CAD [36]. Our results differ from the ones reporting that, in human aortic endothelial cells, rapamycin reduces eNOS expression and activity [37]. These differences might be due to the high heterogeneity of endothelial cells located in different vascular beds [38]. 

In healthy coronary arteries, permeability is very limited and properly regulated [29,30,31]. In inflammation, endothelial permeability barrier function is weakened because of the destabilization of intercellular junctions between endothelial cells, which are crucial in regulating paracellular diffusion [29]. VE-CAD is the main organizer of the opening and closing of the endothelial barrier and is central in permeability changes [39,40,41]. ZO-1 is a tight junction component and its depletion results in tight junction disruption [42]. We found that SIR decreases the total amounts of both ZO-1 and VE-CAD, and this is more accentuated when hCAEC are maintained in 3 mM Mg. Immunofluorescence demonstrates the presence of interendothelial gaps only with antibodies against ZO-1. Surprisingly, when we measured endothelial permeability, we found no marked differences, apart from a modest, albeit significant, increase after treatment with SIR50 in the cells maintained in 3 mM Mg. It is well accepted that the plasticity of the junction is regulated in a very complex way through post-translational modification and protein–protein interactions [43]. Indeed, in human aortic endothelial cells, SIR impairs the barrier function by disrupting the p120 catenin–VE cadherin interaction [23]. Another player that might be involved in modulating endothelial permeability is connexin 43 [44,45]. More experiments are necessary to better define the mechanisms involved in the alteration of the endothelial barrier in our experimental setting.

Endothelial viability and migration are important steps in driving vascular wall healing and re-endothelialization of a scaffold. Subconfluent hCAEC are very sensitive to the detrimental effects of SIR, which, at the same concentrations, does not affect the viability, the morphology, cytoskeletal organization and intracellular Mg^2+^ concentration of confluent cells. SIR markedly inhibits hCAEC migration, as previously reported by Moss et al. [46], and ascribed to the increase of p27, which inhibits RhoA activation. 3 mM Mg exerts no protective effect on hCAEC, in contrast with data obtained in rabbit coronary endothelial cells, where we have shown that concentrations of Mg higher than 1 mM prevent the inhibitory effects of SIR on their migration [12]. This discrepancy deserves more studies at the molecular level to individuate potential strategic mechanisms to counteract SIR inhibitory effect in human cells. 

It is noteworthy that our experiments were performed on healthy hCAEC. It will be interesting to extend these studies also to cell lining stenotic regions of arteries, where hemodynamic stress importantly influences cell morphology and gene expression [47]. 

## 4. Materials and Methods

### 4.1. Cell Culture

Primary hCAEC were purchased from Lonza, cultured in their Endothelial Cells Basal Medium (EBM-2, Lonza, Basel, Switzerland) and expanded for four population doublings according to manufacturer’s instructions. The cells were grown at 37 °C in a humidified atmosphere and at 5% of CO_2_. Many batches of cells were used to overcome possible bias due to individual differences. For survival experiments, cells were plated at 60% (subconfluent) or 100% of confluence in the presence of different concentrations of both SIR and extracellular Mg, while, for all the other experiments, 100% confluent cells were used. In particular, MgSO_4_ was added to the medium containing the physiological concentration of Mg (1 mM) to reach the final concentration of 1.5 mM, 2 mM and 3 mM of Mg. 0 ng/mL (SIR0), 2 ng/mL (SIR2), 10 ng/mL (SIR10), 50 ng/mL (SIR50) and 100 ng/mL (SIR100) of SIR were tested in combination with different concentrations of extracellular Mg. The results were compared to control (1 mM Mg + 0 ng/mL Sir, named thereafter 1 mM Mg SIR0). 

For the adhesion experiments, the U937 cell line (ATCC) was utilized [48]. The cells were cultured in RPMI (Euroclone, Pero, Italy) added with 10% fetal bovine serum, 1% penicillin/streptavidin and 1% L-glutamine, following the manufacturer’s instructions. All the experiments were performed after 48 h of treatment and at least three times.

### 4.2. Viability Assay

To test the effects of the different treatments on cell viability, we used the MTT (3-(4,5-dimethylthiazol-2-yl)-2,5-diphenyltetrazolium bromide) assay. hCAEC were plated at 60% (24,000 cells/cm^2^) or 100% (40,000 cells/cm^2^) of confluence in 96-well plates, and 24 h later we treated them with the different combinations of Mg and SIR. After 48 h of treatment, MTT was added in the culture medium (MTT, 0.5 mg/mL) (Sigma-Aldrich, St. Louis, MO, USA) in a ratio of 1:10 (MTT, 0.05 mg/mL). After 3–4 h at 37 °C, the formazan crystals derived by the degradation of MTT into the mitochondria of living cells were dissolved in EtOH: DMSO (1:1), and absorbance was measured at 550 nm using Varioskan Lux (Thermo Fisher Scientific, Waltham, MA, USA). Data represent the mean ± SD of three separate experiments in triplicate.

### 4.3. Intracellular Mg^2+^ Quantification

The QuantiChrom Magnesium Assay Kit (BioAssay Systems, Hayward, CA, USA) was used to measure intracellular Mg^2+^. The cells were treated for 48 h with the different combinations of Mg and SIR and lysed in PBS. The same amount of lysates (10 μg) was utilized to perform the assay following the manufacturer’s instructions. Data represent the mean ± SD of three separate experiments in duplicate.

### 4.4. In Vitro Wound Assay

hCAEC were seeded on 24-well plates (Greiner bio-one, Frickenhausen, Germany) and cultured in the presence of the different treatments for 48 h. Then, the monolayers were scraped to create a wound in the middle of the well, washed twice with PBS and maintained in starvation media containing Mg and/or SIR for an additional 24 h. Finally, the cells were fixed and colored with Crystal Violet for 20 min, washed with PBS twice and visualized by optical microscope (4× magnification). The images were analyzed using ImageJ. The cell velocity was calculated as the ratio between the edges’ distance and the time, and the results are expressed as the cell velocity ±SD.

### 4.5. Adhesion Assay

hCAEC were seeded in a 24-well plate and treated with Mg and SIR. In parallel U937 cells were incubated with Calcein AM (Thermo Fisher Scientific) following the manufacturer’s instructions to make them fluorescent. 48 h later, hCAEC were activated by a four hours treatment with LPS (final concentration 10 μg/mL). Then, fluorescent-U937 cells were added on top of each well and incubated with hCAEC monolayer for 30 min. After extensive washing with PBS, the adhered cells were fixed in PBS containing 4% paraformaldehyde and 2% sucrose (pH 7.6). To visualize and take pictures of the results, the Floid Microscope (Thermo Fisher Scientific) was utilized, and the attached U937 for each field were counted. The experiment was performed three times, and the data are expressed as the number of cells attached/field ± SD.

### 4.6. Confocal Imaging

To visualize the cytoskeleton, the cells grown on microscope glasses and treated for 48 h were fixed in PBS containing 4% paraformaldehyde and 2% sucrose (pH 7.6), permeabilized with Triton 0.3% and stained with fluorescein fluorescent phalloidin (Thermo Fisher Scientific). 

To stain the adhesion molecules, the cells were fixed, permeabilized and incubated with antibodies anti-VE-CAD or anti-ZO-1 overnight at 4 °C. The cells were then stained with an Alexa Fluor 546 secondary antibody (Thermo Fisher Scientific), and 4′,6-Diamidine-2′-phenylindole dihydrochloride (DAPI, Sigma-Aldrich) was used to stain the nuclei. Finally, the samples were mounted with ProLong™ Gold Antifade Mountant (Thermo Fisher Scientific), and images were acquired using a 40× objective in oil by an SP8 Leica confocal microscope.

### 4.7. Enzyme-Linked Immunosorbent Assay–ELISA

The cells were seeded on 6-well plates and treated for 48 h in the presence of 1 or 3 mM of extracellular Mg with (2 or 50 ng/mL) or without SIR (0 ng/mL). For the quantitative determination of P-selectin (P-SEL), Vascular Cell Adhesion Protein 1 (VCAM-1) (Cloud-clone corp., Katy, TX, USA) and Intercellular Adhesion Molecule 1 (ICAM-1) (Cusabio Technology LLC, Houston, TX, USA), ELISA kits were used according to the datasheet instructions. All the ELISA were performed on 20 µg of cell extracts in triplicate three times, and the data are expressed as the percentage compared to control (1 mM Mg SIR0) ± SD.

### 4.8. Western Blot

hCAEC were lysed in 10 mM Tris-HCl (pH 7.4) containing 3 mM MgCl_2_, 10 mM NaCl, 0.1% SDS, 0.1% Triton X-100, 0.5 mM EDTA and protein inhibitors, separated on SDS-PAGE and transferred to nitrocellulose sheets at 400 mA for 2 h at 4 °C. Western blot analysis was performed using antibodies against ZO-1, VE-CAD (Thermo Fisher Scientific), eNOS (Cell Signaling, Danvers, MA, USA), eNOS phosphorylated at Ser-1177 (P-eNOS, BD Biosciences, New Jersey, USA) and Actin (Tebu Bio-Santa Cruz, Magenta, Italy), which was used as a control of loading. After extensive washing, secondary antibodies labeled with horseradish peroxidase (Amersham Pharmacia Biotech Italia, Cologno Monzese, Italy) were used. Immunoreactive proteins were detected by the SuperSignal chemiluminescence kit (Thermo Fisher Scientific). All the experiments were performed at least three times, and a representative blot is shown. Densitometry of the bands was performed with the software ImageLab (Bio-Rad, Hercules, CA, USA) on at least three blots. Data are expressed as the fold change compared to the control (1 mM Mg SIR0) ± SD.

### 4.9. X-PerT Assay

96-well plates were coated with biotin-fibronectin (20 µg/mL, Cytoskeleton Inc, Denver, CO, USA) for 30 min at RT. Then, hCAEC were seeded on the top and treated with Mg and SIR for 48 h. At the end of the experiment, Avidin-FITC (Thermo Fisher Scientific) was added in the medium (final concentration 2.5 µg/mL) for 2 min and then washed twice with PBS. When spaces are present between the cells, avidin-FITC strictly binds to the coating of biotin-fibronectin and fluorescence can be detected. The fluorescence was read (Ex = 490 nm; Em = 560 nm) using Varioskan Lux (Thermo Fisher Scientific). The experiment was performed three times in triplicate, and the data are expressed as the percentage compared to control (1 mM Mg SIR0) ± SD.

### 4.10. Measurement of Released NO

The cells were treated for 48 h in the presence of SIR in a medium containing either 1 or 3 mM Mg, and at the end of the experiment the culture media was collected. Released NO was assessed by the Griess Assay [49]. Briefly, freshly prepared Griess reagent, made with sulfanilamide and N-(1-naphthyl)ethylenediamine (1:1), was added to the collected culture media. The absorbance was measured at 550 nm using Varioskan Lux (Thermo Fisher), and the amounts of nitrites in the samples were determined using a standard curve generated with known concentrations of sodium nitrite (NaNO_2_). All these experiments were performed three times in triplicate, and the data are expressed as the percentage compared to control (1 mM Mg SIR0) ± SD.

### 4.11. Statistical Analysis

Data are reported as means ± SD. The data were normally distributed and analyzed using one-way repeated measures ANOVA. The p-values deriving from multiple pairwise comparisons were corrected by the Bonferroni method. Statistical significance was defined for *p*-value ≤ 0.05. * *p* ≤ 0.05; ** *p* ≤ 0.01; *** *p* ≤ 0.001.

## 5. Conclusions

This study underscores differences in the response to SIR and Mg in rabbit vs. human cells and points to the importance of experimental studies on cells derived from different species. It also underlines that SIR might retard endothelialization because it inhibits migration and impairs sub-confluent cell viability, while exerting anti-inflammatory actions that might be involved in preventing restenosis and thrombosis. 

## Data Availability

Data can be found on https://dataverse.unimi.it/dataverse/Mg_SIR_hCAEC.

## Supplementary Materials

The supporting information can be downloaded at: https://www.mdpi.com/article/10.3390/ijms24032930/s1.
