# Supplementary material for: The Effects of Sirolimus and Magnesium on Primary Human Coronary Endothelial Cells: An In Vitro Study
Source: Int J Mol Sci. 2023 Feb 2;24(3):2930. doi: 10.3390/ijms24032930 (PMC9917770; doi:10.3390/ijms24032930)
Supplement: Supplementary file 1 [file ijms-24-02930-s001.zip › ijms-2163373-supplementary.pdf]

The Effects of Sirolimus and Magnesium on Primary Human Coronary Endothelial Cells: An In Vitro Study

Giorgia Fedele [1], Sara Castiglioni [1], Jeanette A. M. Maier [1,*] and Laura Locatelli [1]

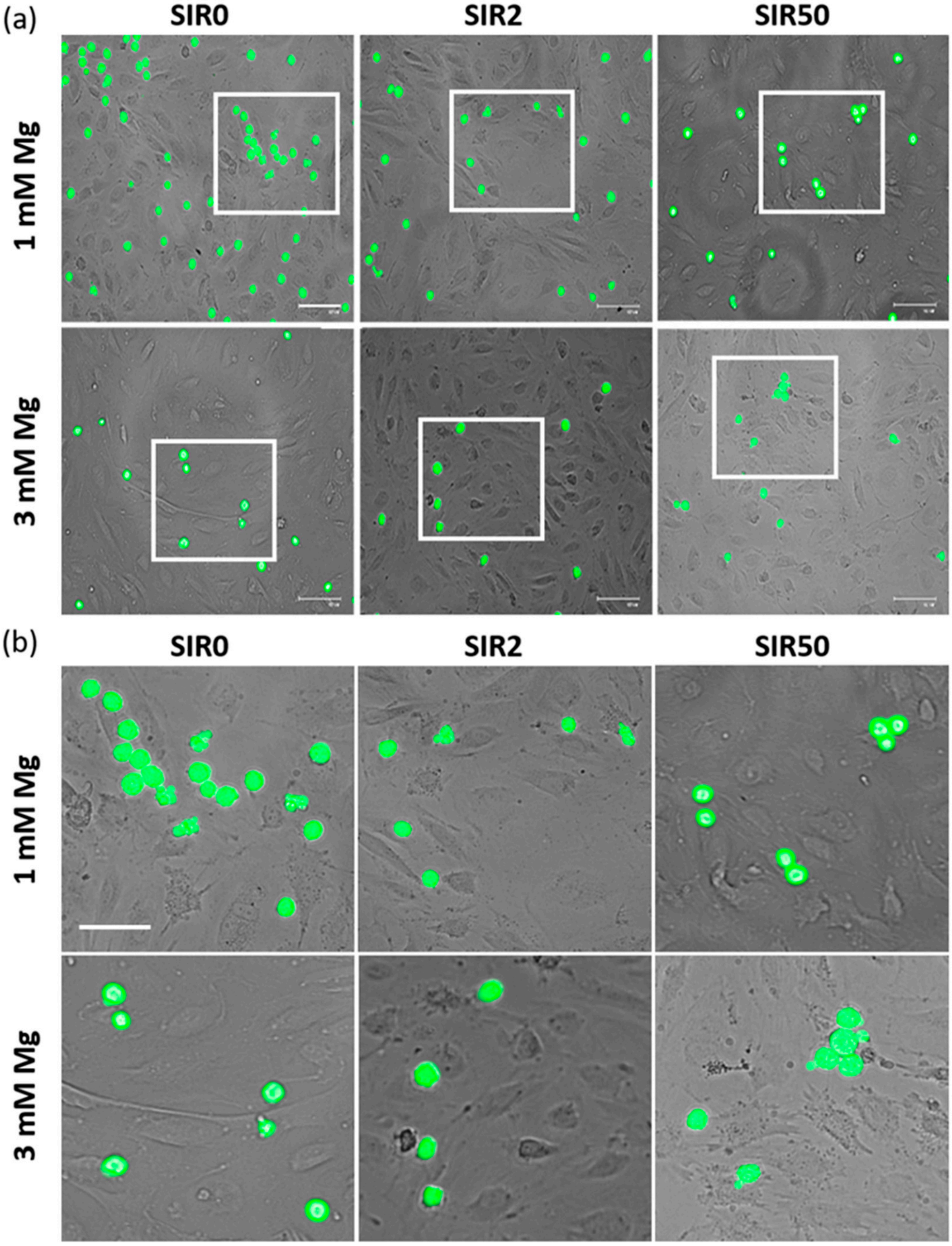

Figure S1. The effects of SIR on the adhesion of U937 to hCAEC. After 4h of treatment with LPS, hCAEC were cultured with 1 or 3 mM Mg in the absence (SIR0) or in the presence of SIR (2 and 50 ng/mL). Fluorescent-U937 cells were incubated with hCAEC for 30 min. (a) Images were acquired using Floid Microscope and the attached U937 for each field were counted. Scale bar: 40μm. (b) Images in "a" were zoomed to better appreciate both hCAEC and U937 cells. Scale bar: 40μm.

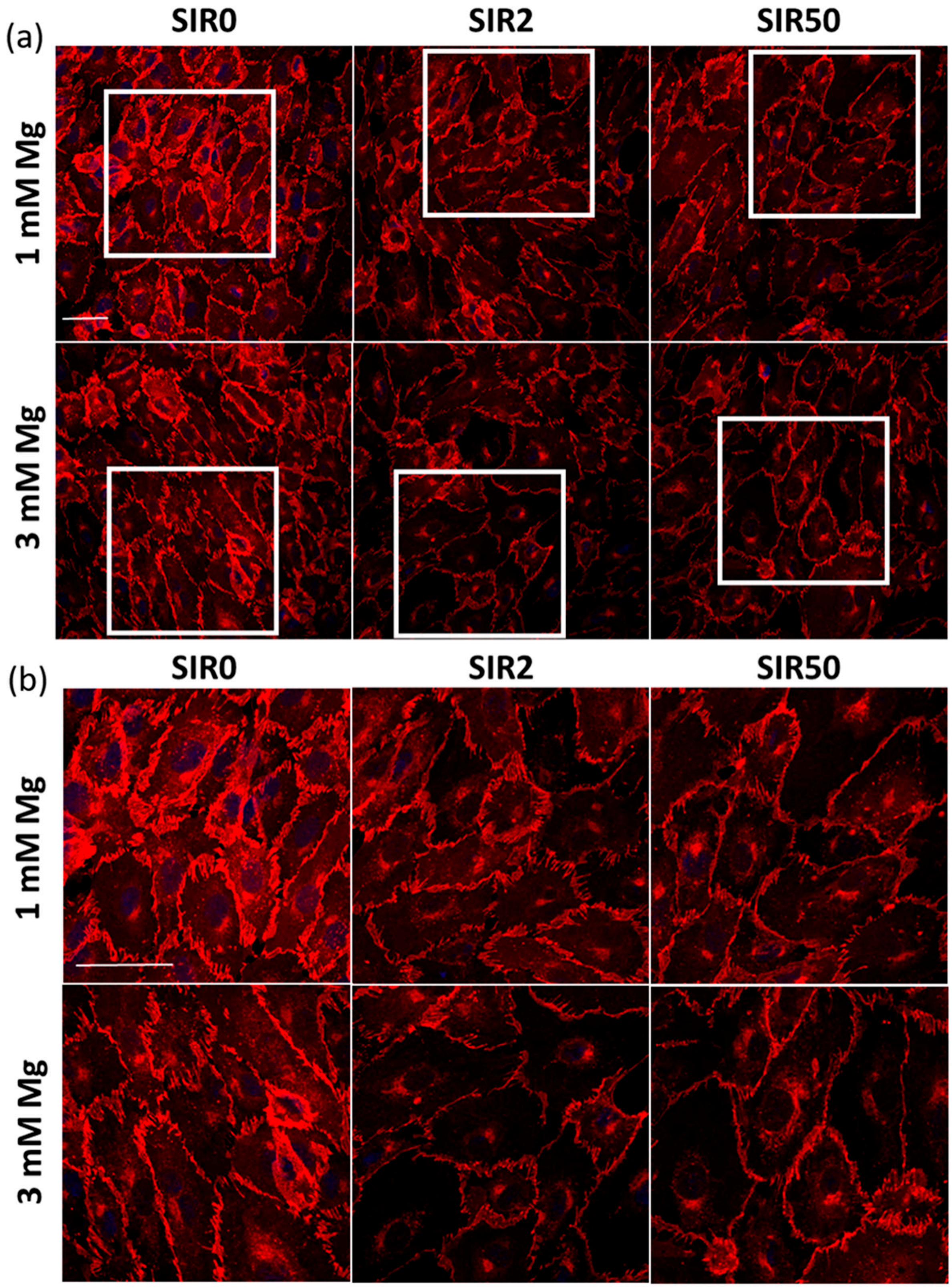

Figure S2. The effects of SIR on the localization of VE-CAD. hCAEC were cultured with 1 or 3 mM Mg for 48h in the absence (SIR0) or in the presence of SIR (2 and 50 ng/mL). The cells were incubated with antibodies anti-VE-CAD and then stained with an Alexa Fluor 546 secondary antibody. DAPI was used to stain the nuclei. (a) Images were acquired using a 40X objective in oil by an SP8 Leica confocal microscope. Scale bar: 40μm. (b) Images in "a" were zoomed to better appreciate the integrity of VE-CAD junctions. Scale bar: 40μm.

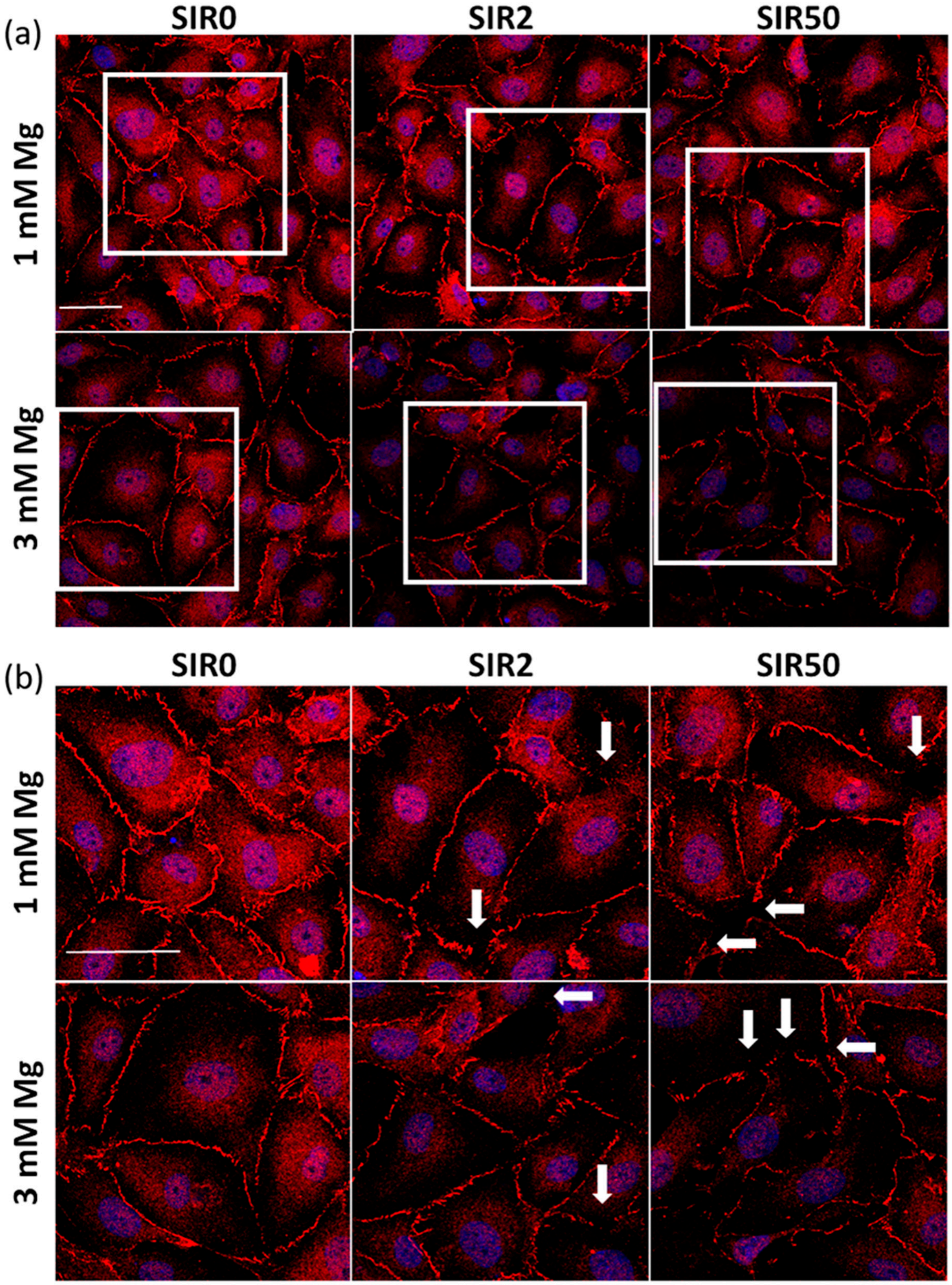

Figure S3. The effects of SIR on the localization of ZO-1. hCAEC were cultured with 1 or 3 mM Mg for 48h in the absence (SIR0) or in the presence of SIR (2 and 50 ng/mL). The cells were incubated with antibodies anti-ZO-1 and then stained with an Alexa Fluor 546 secondary antibody. DAPI was used to stain the nuclei. Images were acquired using a 40X objective in oil by an SP8 Leica confocal microscope. Scale bar: 40μm. (b) Images in "a" were zoomed to better appreciate the integrity of ZO-1 junctions. Scale bar: 40μm.
